# Unraveling the link between frailty and Alzheimer’s disease biomarkers in patients with mild cognitive impairment

**DOI:** 10.1007/s11357-025-01547-3

**Published:** 2025-02-10

**Authors:** Simona Buscarnera, Marco Canevelli, Giuseppe Bruno, Valentina Garibotto, Giovanni Battista Frisoni, Federica Ribaldi

**Affiliations:** 1 Department of Human Neuroscience, Sapienza University of Rome, Viale dell’Università, 30, Rome, 00185 Italy; 2 National Center for Disease Prevention and Health Promotion, Italian National Institute of Health, Rome, Italy; 3 Aging Research Center, Department of Neurobiology, Care Sciences and Society, Karolinska Institutet and Stockholm University, Stockholm, Sweden; 4 Division of Nuclear Medicine and Molecular Imaging, Geneva University Hospitals, Geneva, Switzerland; 5 CIBM Center for Biomedical Imaging, Geneva, 1205 Switzerland; 6 Laboratory of Neuroimaging and Innovative Molecular Tracers (NIMTlab), Geneva University Neurocenter and Faculty of Medicine, University of Geneva, Geneva, 1205 Switzerland; 7 Memory Clinic, Geneva University Hospitals, Geneva, Switzerland; 8 Laboratory of Neuroimaging of Aging (LANVIE), University of Geneva, Geneva, Switzerland

**Keywords:** Alzheimer’s disease, Mild cognitive impairment, Frailty, Biomarkers, Amyloid, Tau

## Abstract

Alzheimer’s disease (AD) can be identified through biomarkers of amyloid (A) and tau (T) pathology. Frailty, a measure of biological aging, could impact the association between AD neuropathology and its clinical manifestation. We aimed to investigate the relationship between frailty and AD biomarkers among people with mild cognitive impairment (MCI) attending a university memory clinic. Data were collected from a cohort of patients with MCI at the Memory Center of Geneva University Hospital (Switzerland). Frailty was assessed using a 35-item Frailty Index (FI). A and T positivity were determined through amyloid and tau PET or CSF analysis. Participants were divided into two subgroups: (i) A + T + (both amyloid and tau positive) and (ii) E/N (either A + or T + , neither A + nor T +), including all other combinations of A/T status. We first explored the correlation between FI, age, and education. Demographics, FI scores, and neuropsychological test results were then compared between these two groups. Logistic regression models, adjusted for age, sex, and education were used to examine the association between FI and AT positivity. One hundred twenty patients were included. FI was positively correlated with age and inversely with education. A + T + patients exhibited lower FI scores compared to E/N participants (0.13 ± 0.10 vs. 0.15 ± 0.08, *p* = 0.01). Logistic regressions found a negative association between FI and A + T + (OR 0.6, 95% CI 0.32–0.90; *p* = 0.02). Frailty is associated with a lower likelihood of AD biomarker positivity in patients with MCI. Frailty might reflect alternative pathophysiological mechanisms contributing to cognitive impairment.

## Background

Alzheimer’s disease (AD) stands as the primary cause of dementia and one of the greatest healthcare challenges of the twenty-first century [1]. Pathologically, AD is mainly characterized by the accumulation of amyloid plaques and tau neurofibrillary tangles which deposit in cortical and subcortical structures leading to synaptic loss and neurodegeneration. Such biological alterations are known to start decades before the clinical manifestations [2].

Although the diagnosis of AD dementia is primarily clinical, there is a growing interest in identifying and using biomarkers to demonstrate its underlying neuropathophysiological changes in vivo. Technological advances are increasingly enabling researchers and clinicians to detect the presence and extent of amyloid and tau pathologies, as well as other co-pathologies, facilitating an earlier etiological definition of neurocognitive disorders. In 2016, aiming to speed up the transition towards a biological definition of AD, the National Institute on Aging and Alzheimer’s Association (NIA-AA) introduced the “A/T/N” research framework, which was further updated in 2024 [3]. This classification categorizes individuals based on AD biomarkers, dichotomized as follows (i.e., positive or negative): “A” for β-amyloid abnormalities (at the amyloid PET scan or CSF Aβ 42 measurement), “T” for tau protein changes (via tau PET scan, or CSF phospho-tau assessment), and “N” for evidence of neurodegeneration (through [18F]-fluorodeoxyglucose–PET, structural MRI, or CSF total tau determination).

However, there is a significant variability between the clinical manifestations, the progression of the disease, and its neuropathological hallmarks (i.e., biomarker changes). For example, about 30 to 40% of older adults may show neuropathological changes at autopsy without having experienced any cognitive dysfunctions [4–6]. Conversely, other individuals with similar levels of AD neuropathology may exhibit more severe clinical and cognitive symptoms [7]. This discrepancy between AD-related lesions and their phenotypic expression remains largely unexplained and has been mostly attributed to concomitant neuropathological changes (e.g., Lewy body disease and other proteinopathies, vascular damage, neuroinflammation) [8]. However, it may be hypothesized that additional factors, not directly linked to brain damage but more accurately reflecting the multisystemic aging process, may influence cognition and contribute to the observed clinical-pathological mismatch [9]. Particularly, frailty is drawing significant interest in this context.

Frailty is defined as a multisystemic clinical syndrome characterized by the reduction in the ability to respond to stressors, which increases the risk of adverse health outcomes [10]. Frailty is commonly measured through the Frailty Index (FI), a measure that bases its foundations on the deficit accumulation model [11]. According to this model, an individual’s frailty status is proportional to the degree of accumulation of health deficits over the life course. Frailty, as deficit accumulation, is increasingly investigated in many medical fields to tentatively capture the clinical and biological heterogeneity of aging-related conditions [12–14].

In particular, the association between frailty and dementia has increasingly been investigated. Frailty has consistently emerged as an independent predictor of incident dementia [15, 16]. Moreover, it has preliminarily been shown to moderate the association between AD neuropathology, candidate biomarkers, and their clinical manifestation [17, 18]. Additionally, there is growing recognition of frailty’s potential role in the prognostic assessment of individuals with cognitive disturbances [19]. To date, no study has explored the influence of frailty AD biomarker status in a clinical setting. Exploring this relationship could yield significant insights into the pathophysiological mechanisms of AD. Moreover, it may help ascertain whether frailty could serve as a valuable clinical tool for predicting the biomarker status of patients with cognitive disturbances.

The present cross-sectional study aimed to investigate the potential association between frailty and AD biomarkers among individuals with mild cognitive impairment (MCI).

## Methods

### Population and setting

Study participants were drawn from the dataset of the Memory Center (CdM) of the Geneva University Hospitals (Switzerland). The CdM is a tertiary memory clinic dedicated to the diagnosis, prognosis, and treatment of neurocognitive disorders, where patients are evaluated by a multidisciplinary group of experts, including neurologists, neuropsychologists, neuroradiologists, and nuclear medicine physicians.

Participants were considered eligible for inclusion in the analysis if they (i) had received a diagnosis of MCI, (ii) had the 35 variables needed to compute a FI at their baseline (with a maximum number of missing values of 7.5%), (iii) had available PET or CSF data to classify their amyloid and tau biomarker status, and (iv) had available data on covariates (age, sex, and education level). MCI was defined based on the following clinical criteria: (i) cognitive concern reported by the patient and/or informant (i.e., family member or close friend); (ii) objective evidence of cognitive impairment, based on a comprehensive neuropsychological test battery; and (iii) no functional impairment in daily living activities [20]. The Mini-Mental State Examination (MMSE) was used to assess global cognition. Data on the APOE status of participants was also considered, when available.

### Frailty index

A 35-item FI was calculated by following a standard procedure [21], considering negative (i.e., health deficits), age-related, and multidimensional health variables collected during both clinical and research visits. Overall, the index included 22 health conditions and laboratory tests, along with 13 clinical signs and symptoms (Table 1). All binary variables were recoded as “0” for “absent” or “normal” and “1” for “present” or “abnormal.” For ordinal and continuous variables, standardized thresholds were used. FI was calculated as the ratio of the sum of deficits presented by each individual and the total number of considered variables (i.e., 35). Neuropsychiatric disturbances and functional impairments were not considered in the computation of the FI to prevent any overlap with the diagnosis of MCI/dementia.

**Table 1:** Frailty Index with 35 items. The index is expressed as a ratio of deficits present to the total number of deficits considered (i.e.,35)

	Variables	Score
1	Cardiovascular diseases	Absent = 0, present = 1
2	Atrial fibrillation	
3	Dermatologic diseases	
4	Respiratory diseases	
5	Head diseases (hear, nose, throat)	
6	Hepatic diseases	
7	Connective tissue diseases	
8	Muscular diseases	
9	Osteoarticular diseases	
10	Thyroid diseases	
11	Other endocrine diseases	
12	Hematopoietic-lymphatic diseases	
13	Genital diseases	
14	Urinary diseases	
15	Gastrointestinal diseases	
16	Diabetes	
17	Malignancies	
18	Dyslipidemia	
19	Hypotension	
20	Hypertension	
21	Medications	< 5 = 0; ≥ 5 = 1
22	Hemoglobin	Normal = 0, abnormal = 1
23	Hearing loss	Absent = 0, present = 1
24	Tremor	
25	Pain	
26	Vertigo	
27	Bradykinesia	
28	Walking Impairment	
29	Balance Impairment	
30	Urinary incontinence/urgence	
31	Falls	
32	BP Diastolic	< 90 mmHg = 0 ≥ 90 mmHg = 1
33	BP Systolic	< 140 mmHg = 0 ≥ 140 mmHg = 1
34	Heart rate	< 100 and > 60 bpm = 0 > 100 or < 60 bpm = 1
35	BMI	< 30 and > 18.5 = 0 ≥ 30 or < 18.5 = 1

### Amyloid and tau PET

A subgroup of participants underwent PET scans to detect the presence of amyloid and/or tau pathology, with 64 patients receiving amyloid PET scans and 79 receiving tau PET scans. Amyloid-PET images were acquired using 18F-Flutemetamol, 18F-Florbetapir, and tau-PET images were acquired using 18F-Flortaucipir, reconstructions at the Nuclear Medicine and Molecular Imaging Division of the Geneva University Hospitals using a Siemens Biograph mCT or Vision PET scanner, using tracer-specific protocols [22]. 18F-Florbetapir images were acquired 50 min after the intravenous administration of approximately 200 Mbq of the radiotracer, for 15 min. 18F-Flutemetamol images were acquired 90 min after the injection of approximately 150 MBq of radiotracer for 20 min. 18F-Flortaucipir images were acquired 75 min after the injection of 180 MBq for an acquisition time of 30 min. For all images, we used a 3D OSEM iterative reconstruction (4 iterations, 12 subsets on the mCT scanner and 4 iterations, 5 subsets on the Vision scanner), corrected for randoms, dead time, normalization, scatter, attenuation, and sensitivity.

Amyloid status (i.e., A + vs. A −) and tau status (i.e., T + vs. T −) were defined based on standardized expert visual assessment (https://www.ema.europa.eu/en/documents/product-information/tauvid-epar-product-information_en.pdf).

### Cerebrospinal fluid

When PET was not available, the presence of amyloid and tau pathology was assessed through CSF biomarkers. CSF Aβ42 and p-tau 181 were used adopting different cutoff values for positivity depending on the specific analysis machine employed. Specifically, a CSF Aβ42 level lower than 780 pg/ml with Innotest [23] or 720 pg/ml with Lumipulse was considered indicative of A positivity. CSF values p-tau 181 higher than 60 pg/ml with Innotest or 56.5 pg/ml with Lumipulse were instead used to define T positivity. CSF biomarker positivity was determined using cohort-specific thresholds.

### Classification of participants

Two main subgroups of participants were defined based on their biomarkers profiles resulting from the PET or CSF assessment: (i) A + T + , including both amyloid and tau positive patients, and (ii) E/N, including all the other combinations of biomarker’s status (i.e., A − T − , A + T − , A − T +). Based on the A/T/N framework, A + T + status defines AD diagnosis. By following a similar approach, participants were also grouped according to their A (i.e., A + vs. A −) and T (i.e., T + vs. T −) biomarker status.

### Statistics

The statistical analyses were conducted using R Studio version 2023.06.2 (Build 561). We used descriptive statistics to summarize the characteristics of the study population. Continuous variables were reported as mean and standard deviation; categorical variables were reported as frequency and percentage. Spearman’s coefficients were used to measure the direction and strength of the correlation between FI, age, and education. The Mann–Whitney test and the chi-square test were employed to assess differences in demographics, FI scores, and neuropsychological test results between A + T + and E/N patients, as appropriate.

Logistic regression models, adjusted by age (continuous variable), education (continuous variable), and sex (binary variable), were used to explore the relationship between FI scores and the AT status (bivariate dependent variable of interest). Similar statistical models were conducted separately using A and T positivity as bivariate dependent variables of interest. The results were reported as odds ratio (OR) and 95% confidence intervals (95% CI). In these models, FI scores were multiplied by 10 so that OR could be meaningfully interpreted as the change in the risk of having a given biomarker status per 0.1 increase in Frailty Index score (i.e., additional 3–4 deficits). Statistical significance was set at *p* < 0.05. 

## Results

Overall, 120 patients with MCI were considered in the analysis. Among them, 95% had amnestic MCI, and 3% had non-amnestic MCI, whereas the remaining patients were not classified under a specific syndrome. The mean age of the whole sample was 70.3 ± 8.1 years, and 49 (41%) were women (Table 2).

**Table 2:** Demographic, clinical, and neuropsychological characteristics of the whole sample of MCI memory clinic patients and stratified by AT positivity. Data are shown as mean ± standard deviation or *n* (%)

Variables	Whole sample (*n* = 120)	AT subgroups		
		E/N (*n* = 68)	A + T + (*n* = 52)	*p*-value*
Age, years	70.3 ± 8.1	68.7 ± 8.9	72.9 ± 5.7	**0.02**
Education, years	13.4 ± 3.8	13.2 ± 3.8	13.8 ± 3.9	0.43
Sex (F)	49/120 (41%)	30/75 (40%)	19/45 (42%)	0.96
Frailty Index	0.14 ± 0.09	0.15 ± 0.08	0.13 ± 0.10	**0.01**
APOE 𝜀4 carriers	44/105 (42%)	19/66 (29%)	25/39 (64%)	**< 0.001**
MMSE^a^	26.5 ± 2.6	27.1 ± 2.4	26.6 ± 2.6	**0.001**
Clock drawing test	8.4 ± 2.0	8.5 ± 1.8	8.0 ± 2.3	0.40
CSF^b^ Aβ42	895 ± 464	1084 ± 65	534 ± 125	**< 0.001**
CSF p-tau	69 ± 38	53 ± 30	99.1 ± 34.9	**< 0.001**
PET^c^ amyloid positivity	38/64 (59%)	11/37 (30%)	27/27 (100%)	**< 0.001**
PET tau positivity	30/70 (42%)	3/43 (7%)	27/27 (100%)	**< 0.001**

^a^Mini-Mental State Examination, ^b^cerebrospinal fluid, ^c^positron emission tomography

Variables and number of missing values: APOE 𝜀4 carriers, 15; HADS, 2; PET amyloid positivity, 56; PET tau positivity, 50; CSF Aβ42, 24; CSF p-tau, 24

*p-values refer to the Mann–Whitney test for continuous variables and chi-square test for categorical variables

Data in bold emphasis indicate significant *p* value

The FI demonstrated a characteristic right-skewed distribution, ranging between 0 and 0.48, and a median score of 0.14 ± 0.09. FI scores exhibited a statistically significant, positive correlation with age (rho = 0.3, *p* < 0.01), and an inverse correlation with education (rho = − 0.2, *p* = 0.03).

**Fig. 1:**
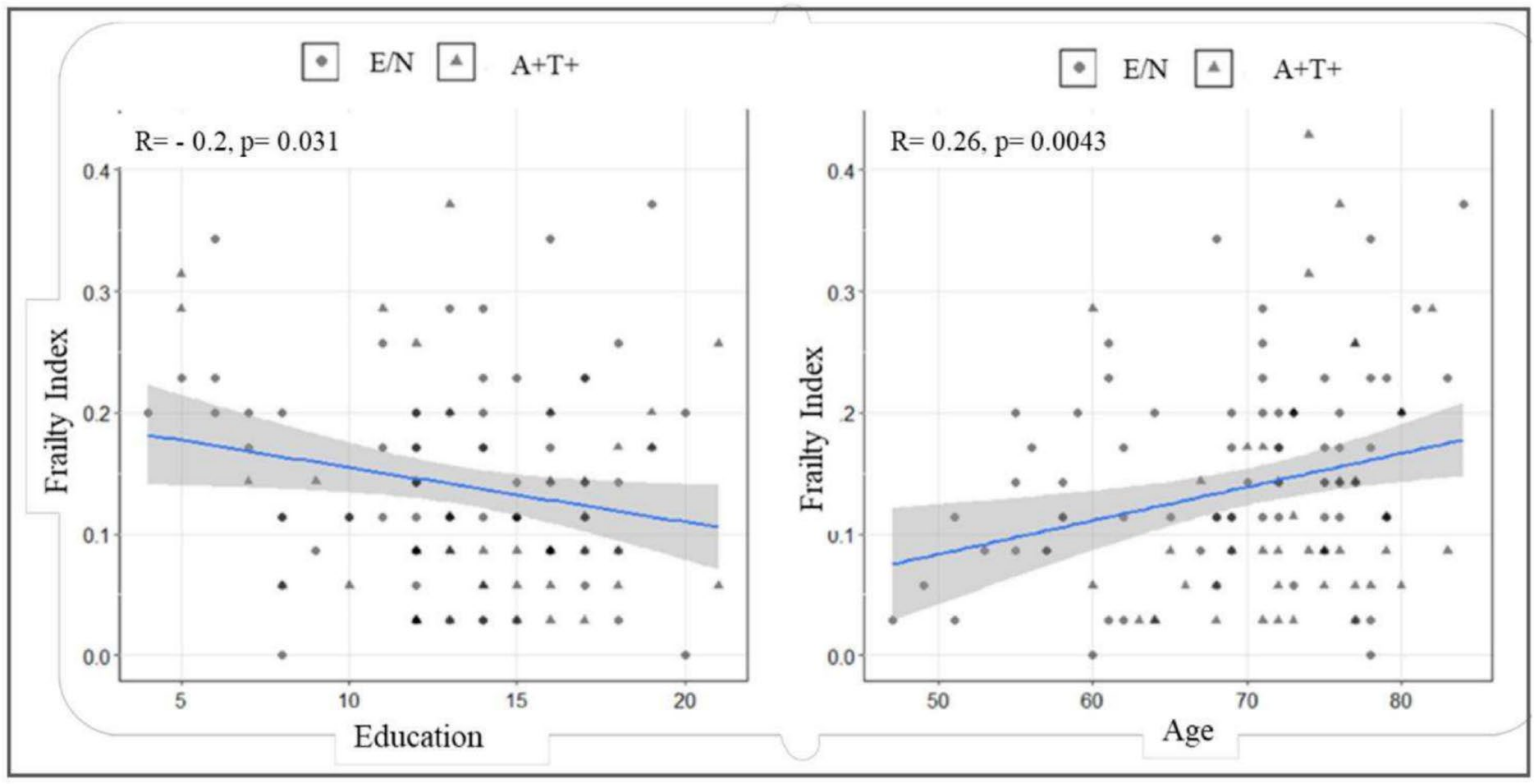
Frailty Index and demographics. FI scores exhibited an inverse correlation with education (rho = − 0.20, *p* = 0.03) and a statistically significant, positive correlation with age (rho = 0.26, *p* < 0.01)

At the univariate analyses, A + T + patients were found to be older (72.9 ± 5.7 vs. 68.7 ± 8.9 years, *p* = 0.02), were more often APOE𝜀4 carriers (64.1% vs. 28.8%, *p* < 0.001), and exhibited lower MMSE (26.6 ± 2.6 vs. 27.1 ± 2.4, *p* = 0.001) and FI scores (0.13 ± 0.10 vs. 0.15 ± 0.08, *p* = 0.01) relative to E/N participants (Table 2 and Fig. 1). Lower FI scores were observed also in A + vs. A − (0.13 ± 0.1 vs 0.15 ± 0.1, *p* = 0.04) and T + vs. T − patients (0.13 ± 0.1 vs 0.15 ± 0.1, *p* = 0.03) (data are not shown).

In a logistic regression model, adjusted by age, sex, and education, frailty was found to be inversely associated with A + T + status.

**Fig. 2:**
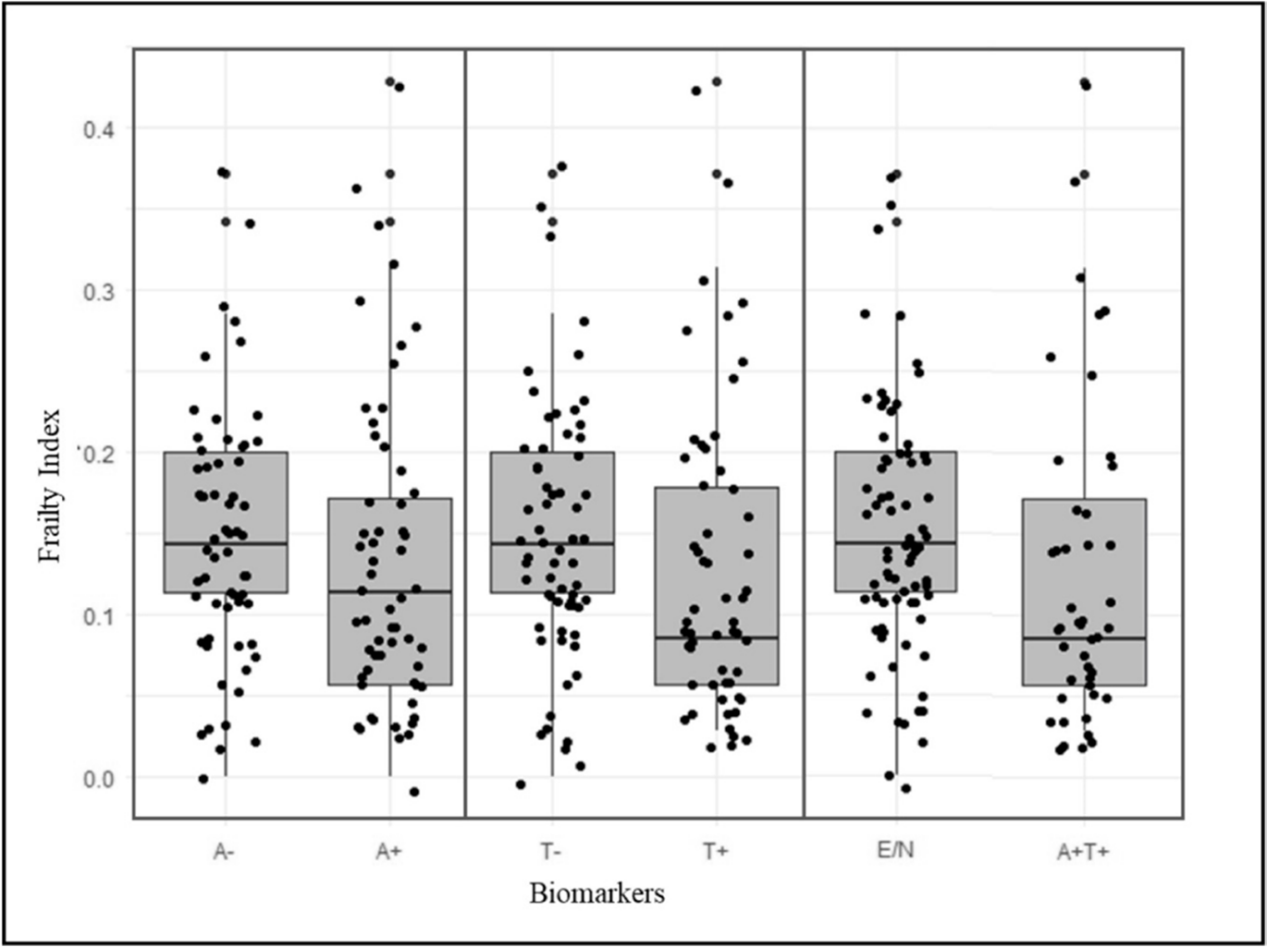
Frailty Index stratified by biomarkers profile. Lower FI scores were observed in A + vs. A − (0.13 ± 0.10 vs 0.15 ± 0.07, *p* = 0.04), T + vs. T − patients (0.13 ± 0.10 vs 0.15 ± 0.08, *p* = 0.04, and A + T + vs E/N status (0.13 ± 0.10 vs. 0.15 ± 0.08, *p* = 0.01)

Each 0.1 increase in FI scores was associated with 40% lower odds of resulting A + T + (OR 0.6, 95% CI 0.32–0.90; *p* = 0.02) (Table 3). Similar findings were obtained when A and T status were separately explored as bivariate dependent variables (Appendix Tables 4 and 5), (Fig. 2).

**Table 3:** Logistic regression model, adjusted by age, sex, and education, exploring the association between Frailty Index and AT status (bivariate dependent variable of interest)

A + T + vs E/N	OR	95% CI	*p*-value
Frailty Index (per 0.1 increase)	0.6	0.3–0.9	**0.02**
Age (years)	1.1	1.0–1.2	** < 0.01**
Education (years)	1.0	0.9–1.1	0.93
Sex (male)	1.0	0.5–2.3	0.93

Data in bold emphasis indicate significant *p* value

## Discussion

In the present study, we explored the association between frailty and the biomarker status of patients with MCI referred to a memory clinic. Overall, we found that higher frailty degrees were associated with lower odds of AT positivity. Accordingly, higher FI scores reduced the likelihood of amyloid and tau positivity when considered individually.

Previous studies have investigated frailty among individuals with MCI, showing that frailty heightens the risk of conversion to dementia and reduces the probability of more favorable cognitive transitions (e.g., reversion to normal cognition) [24–26]. If confirmed, the present findings may suggest that measuring frailty could help disentangle the pathophysiological and clinical complexity of AD. For instance, its clinical assessment may provide useful information on the likelihood of resulting positive to AD biomarkers at the MCI stage. This may have important implications in terms of resource allocation [27]. Indeed, in clinical practice, frailty assessment could help identify those MCI cases in which it may be appropriate to proceed with biomarker assessment considering the expected positivity or negativity. In a clinical trial setting, this information could be valuable in reducing screening failure rates.

Two main, complementary hypotheses can be proposed to tentatively explain the observed negative association between frailty and AD biomarker status. The first hypothesis originates from the conception of frailty as a condition of exhausted homeostatic reserves [28]. This loss of reserve may influence the ability to tolerate the progressive accrual of neuropathological lesions and, thus, their phenotypic expression. As suggested by previous clinical-pathological studies, frailty weakens the relationship between AD pathology and dementia [17]. That is, at high degrees of frailty, even a minimal burden of brain lesions may result in overt cognitive deficits. The same load of neuropathology is instead well tolerated by robust individuals and may remain subclinical. Similar findings were obtained considering AD biomarkers and polygenic risk. Among individuals with similar biomarker changes (e.g., high amyloid burden at the PET scan) or similar polygenic risk, worse frailty increases the risk of MCI and dementia [18, 29]. The second hypothesis deals with the multifactorial nature of cognitive disorders, that are increasingly recognized as multiply determined conditions [30]. It may be postulated that frailty captures alternative aging-related pathophysiological mechanisms that, synergistically with amyloid and tau pathology, contribute to cognitive impairment. In this regard, frailty has repeatedly been associated with different hallmarks of the aging process that have been, in turn, linked with AD and dementia [31–33]. Based on these two hypotheses, the negative association between frailty and AD documented by our study could depend on the fact that (1) in frailer patients, even low amounts of amyloid and tau (lower than the cutoffs used in clinical practice) may result in MCI, and/or (2) in frail patients, other pathophysiological mechanisms (beyond amyloid and tau) may play a central role in the pathogenesis of cognitive dysfunctions.

It is noteworthy that frailty seems to relate to the AD biomarker status of MCI patients in the opposite direction to chronological age, which instead appears to increase the probability of being affected by AD pathology. This reinforces the notion that age does not fully capture the biological and phenotypic complexity of aging-related diseases and does not support age as a criterion for guiding the clinical use of biomarkers, as instead proposed in recent recommendations [3]. In this context, frailty as a measure of biological aging could complement useful information for the biological profiling of the subject.

Some limitations of the study must be acknowledged, such as the limited sample size and the poor representativeness of the sample, mostly composed of highly educated, European older people attending a Swiss tertiary memory clinic. The study sample primarily comprised participants who were relatively healthy/robust or only slightly frail. This conclusion is supported by a median FI score that was significantly below the established cutoff of 0.25, which is used to identify frailty [34]. As a result, the study fails to provide a definite understanding of the relationship between frailty and AD biomarkers. Based on these considerations, our findings must be confirmed in larger and more diverse populations of older and frailer individuals with MCI attending services in different care settings. Moreover, the cross-sectional design does not allow us to infer causality in the observed associations. Longitudinal studies are needed to explore the interplay of frailty and AD biomarkers in the pathogenesis of MCI and dementia. A major strength of the study is instead having built a FI from variables that are widely and easily collected in routine clinical practice. This increases the transferability of the results. Indeed, having an easy-to-use and reliable tool could provide significant advantages in guiding the patient’s journey toward the most appropriate diagnostic and therapeutic options.

The new landscape of AD research is increasingly focusing on the neuropathological substrates of the disease, such as amyloid and tau. However, based on an increasing amount of evidence, these factors seem to be insufficient to fully explain the occurrence, severity, and trajectory of the clinical manifestations. In this scenario, frailty can be a valuable metric for elucidating the role of the aging process in the pathophysiology and clinical expression of AD and clarifying the role and accuracy of candidate biomarkers. Future studies are needed to confirm whether the FI can be a useful tool for the implementation of a biomarker-based AD diagnosis in clinical settings and as an enrichment criterion in the conduction of clinical trials.

## Data Availability

anonymized data used in this study are available upon reasonable request from the corresponding author.

## Appendix

**Table 4:** Logistic regression model, adjusted by age, sex, and education, exploring the association between Frailty Index and A status (bivariate dependent variable of interest)

A+/A-	OR	95% CI	*P* value
Frailty index (per 0.1 increase)	0.6	0.4–1.0	**0.04**
Age	1.1	1.0–1.2	**0.001**
Education	1.0	0.9–1.1	0.7
Sex (male)	0.7	0.3–1.6	0.4

**Table 5:** Logistic regression model, adjusted by age, sex, and education, exploring the association between Frailty Index and T status (bivariate dependent variable of interest)

T+/T-	OR	95% CI	*P* value
Frailty index (per 0.1 increase)	0.6	0.4–1.0	**0.03**
Age	1.1	1.0–1.1	**0.01**
Education	1.0	0.9–1.1	0.77
Sex (male)	1.3	0.6–2.8	0.53
